# Development and validation of a behavioral index for adaptation to lyme disease

**DOI:** 10.1186/s12889-020-09535-2

**Published:** 2020-09-21

**Authors:** Pierre Valois, David Bouchard, Cécile Aenishaenslin, Denis Talbot, Catherine Bouchard, Sandie Briand, Maxime Tessier

**Affiliations:** 1 Faculty of Education, Université Laval, 2320, rue des Bibliothèques, Quebec City, QC G1V 0A6 Canada; 2 Groupe de recherche en épidémiologie des zoonoses et santé publique (GREZOSP), Faculté de médecine vétérinaire (FMV), Université de Montréal, Saint-Hyacinthe, QC Canada; 3 Faculty of Medicine, Université Laval, 1050 Avenue de la Médecine, Quebec City, QC G1V 0A6 Canada; 4 Public Health Risk Sciences Division, National Microbiology Laboratory, Public Health Agency of Canada, Saint-Hyacinthe, QC Canada; 5 National Public Health Institute of Québec, Montreal, QC Canada

**Keywords:** Adaptation, Lyme disease, Theory of planned behavior, Index, Validation

## Abstract

**Background:**

Recent evidence suggests that climate change and other factors are leading to the emergence of Lyme disease in the province of Quebec, where it previously did not exist. As risk areas expand further north, the population can adopt specific preventive behaviors to limit chances of infection. The objectives of this study were to (1) create an index of Lyme disease prevention behaviors (LDPB), and (2) use the theory of planned behavior (TPB) to explain the decision-making process of people who choose to adopt LDPB.

**Methods:**

A sample of 1959 adults living in a Lyme disease risk area completed a questionnaire by phone (*n* = 1003) or on the Web (*n* = 956). The questionnaire measured whether they did or did not adopt the LDPB proposed by public health officials. It also measured some TPB variables, including their attitude or perceived social norms regarding LDPB.

**Results:**

Our findings led to the creation of a Lyme disease prevention index consisting of 10 behaviors, down from the 19 behaviors initially considered for inclusion in the index. Rates of adoption of each behavior varied tremendously, from 4.30 to 83.80%. All variables of the TPB model (attitude, social norms, and perceived control) were significantly associated with intention to adopt preventive behaviors. Intention itself was significantly associated with adoption of LDPB. Likewise, risk perception was positively correlated with the adoption of LDPB.

**Conclusions:**

This study led to the creation of a Lyme disease prevention index that can be used by public health agencies, researchers, and professionals to monitor the evolution over time of individuals’ LDPB adoption rates. It also showed the usefulness of the TPB in understanding the adoption of LDPB and how intention to adopt such behaviors is formed.

## Background

Floods and heat waves are some of the most common issues linked to climate change, with current predictions showing an increase in their frequency and intensity in the near future [1–4]. However, the impacts of our changing climate on human health include not only these dangerous climatic events, but also an increase in zoonotic diseases, such as Lyme disease. Linked to a specific species of ticks in eastern and Midwestern North America, *Ixodes scapularis*, Lyme disease is slowly gaining ground further north, being found in areas where it used to be completely absent [5–12]. These areas include the province of Quebec, Canada, where the number of confirmed cases of Lyme disease rose from 32 in 2011 to 329 in 2017 [13]. This increase can be partly explained by warmer winters, rising global temperature, more rain, higher humidity, and changing patterns of seasonal weather caused by climate change making those previously inhospitable areas more favorable for *Ixodes scapularis* ticks and their hosts. These factors have led to improved survival and reproduction rates of ticks and their hosts, as well as an extended seasonal risk period [10, 14–17].

Since it was first identified in North America in the 1970s, Lyme disease has become the most commonly reported zoonotic disease in temperate areas [6, 18–20]. Infected *Ixodes scapularis* ticks can transmit *Borrelia burgdorferi*, the bacteria causing Lyme disease, to humans and some mammals. It can cause a variety of symptoms in humans, beginning with the most common: a red expanding or bulls-eye rash surrounding the bite. Present in 70 to 80% of infections, this rash can also be accompanied by fever, exhaustion, headaches, neck stiffness, and muscle and joint pain. Untreated, Lyme disease can then cause a wide variety of multisystemic issues, from arthritic joint deterioration to cardiac or neurological problems.

Fortunately, it is possible to reduce the risks of infection from Lyme disease, even in endemic areas. It has been shown that adopting simple behaviors like checking for ticks after going outside, wearing long clothes that cover the skin or using appropriate tick repellent spray can help prevent infection [21–24]. However, for people living in at-risk areas to implement these actions, they need to be aware of them and consider them effective [25]. Our goal was thus to produce a behavioral index of adaptation to Lyme disease, documenting adoption rates of a wide variety of adaptive behaviors throughout at-risk populations in the province of Quebec. Indices are an ideal way to monitor changes in behaviors throughout the years. Therefore, with the rapid increase of at-risk areas in the province of Quebec and the other provinces in Canada, subsequent studies using such an index could help decision makers better target vulnerable populations for awareness work, as well as provide a better picture of Lyme disease awareness in the population.

Some researchers [26–29] have developed behavioral measures of adaptation to Lyme disease based on scientific knowledge of what would constitute a good adaptation of individuals. However, these measures have never been tested with sophisticated modern psychometric analyses (e.g. item analysis based on item response theory, confirmatory factor analysis). The objectives of the present study were to start from the behavioral scales developed by these authors (1) to create and validate an index of Lyme disease prevention behaviors (LDPB) and (2) to use the theory of planned behavior (TPB) to explain the decision-making process of people who choose to adopt LDPB. In fact, the TPB will be useful for estimating the nomological validity of the index, and it will be well suited for identifying the social cognitive factors (e.g. social pressure, perceived barriers) that can be used to inform interventions designed to change Lyme disease prevention behaviors.

### Theory of planned behavior

The TPB is a theoretical model that has already been successfully used to explain various behaviors related to the environment, such as adoption of climate change adaptation behaviors [30, 31], support for climate change policies [32], environmental sustainability through recycling [33], and use of improved natural grassland [34]. One meta-analysis has shown the effectiveness of the TPB for developing and conducting behavior change interventions [35].

**Fig. 1:**
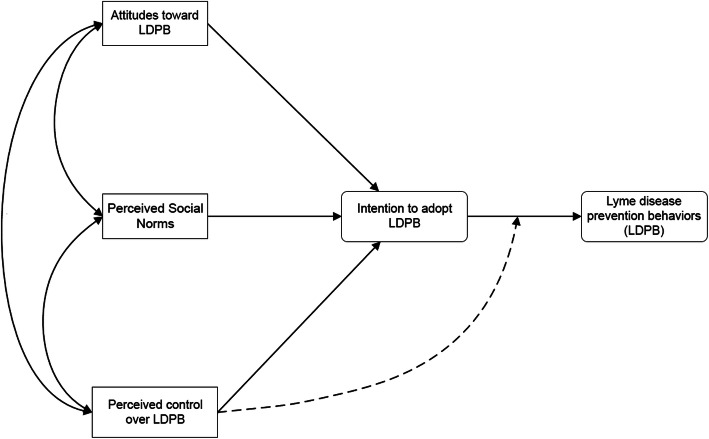
The theory of planned behavior model

This theoretical framework (see Fig. 1) postulates that intentions to perform LDPB and perceived behavioral control (i.e. people’s perceptions of their ability to perform LDPB and/or the presence of factors that may facilitate or impede performance of LDPD) are the immediate antecedents of LDPB, and that perceived control can have a direct effect on behavior while also influencing behavior indirectly through its effect on intentions.

According to Fishbein and Ajzen [36], the effect of intention on action should be stronger when actual control is high rather than low. For instance, when people believe that they actually have control over the adoption of LDPB, they tend to act in accordance with their intentions. Conversely, if they believe that they have low control over the adoption of LDPB, they do not tend to act in accordance with their intentions [37].

The TPB also postulates that people’s intention to adopt LDPB should increase to the extent that they hold favorable attitudes toward such behaviors, think that significant others support the performance of these behaviors (i.e. perceived social norms), and perceive that they have control over them.

Finally, the TPB proposes that various background factors (e.g. gender, education, personality) can have primarily indirect effects on intentions and on adoption of LDPB through attitudes, perceived norm, and perceived control. Thus, to gain further insight into the underlying reasons for the implementation of LDPB among Quebec’s at-risk population, we examined the potential impact of two background factors from the health belief model: perceived risk or vulnerability represented by the disease, and perceived impact of the disease (severity) on health [38, 39].

## Methods

### Participants

A polling firm surveyed 1959 individuals (women, 62.02%), of whom 1003 (women, 68.30%) were contacted by telephone and 956 (women, 55.44%), through the online questionnaire. Cochran’s formula [40] was used to estimate the sample size with a 95% confidence level, a maximal variance of 4 for a five-point scale, and a precision level of 0.12.

All respondents came from a specific subset of Quebec municipalities considered to be at a low to high risk for Lyme disease [41–43], in the regions of Estrie, Montérégie, Mauricie-et-Centre-du-Québec, Outaouais, Lanaudière, Montréal, Laurentides, and Chaudière-Appalaches. Quotas by region were specified to the polling firm. They were adjusted using the Kish selection method [44, 45], with a maximum set for the most populous region, Montreal, to ensure that the sample from each region was sufficient and still representative.

Respondents had to be at least 18 years old to participate and the age distribution of the participants was: 18–34 [8.32%], 35–44 [14.09%], 45–54 [18.33%], 55–64 [27.05%], 65–74 [22.61%], 75 and over [9.29%]. They could complete the questionnaire and the interviews in French or in English. Most spoke French (91.88%) and were born in the province of Quebec (89.84%). Their highest education levels were an elementary school diploma (10.11%), a high school diploma (29.50%), a college or professional diploma (26.60%), or a university degree (33.13%). Regarding the composition of their household, 23.84% lived alone, 42.42% lived with another person, and 32.77% lived with two or more other persons.

The household structure consisted of a couple without children in 36.40% of the cases; a couple with children for 32.21% of them; and other structures, including living alone or with a roommate, for 30.12% of the respondents. The household income was less than CAD$20,000 for 8.73%, between CAD$20,000 and CAD$60,000 for 32.21%, between CAD$60,000 and CAD$100, 000 for 20.62%, and over CAD$100, 000 for 20.21%.

### Data collection

The data were collected from May 18, 2018, to August 28, 2018, a period during which ticks nymphs are more active. The telephone interviews took an average of 20 min, and the response rate was 24.5%. A maximum of 10 attempts were made to establish contact before rejecting a telephone number. For the online questionnaire, a web panel that had been built by the polling firm prior to this data collection was used. Only the panelists living in the target areas were contacted by email, and their municipalities of residence were verified a second time with a question in the online questionnaire. The online questionnaire took an average of 17 min to complete, and the response rate was 17%.

### Measures

#### Index of Lyme disease prevention behaviors (LDPB)

To identify an initial list of preventive behaviors, 19 items were selected based on a literature review [21, 22, 25, 46–48] and recommendations from public health agencies [42, 49]. These items are listed in Table 1. Filter questions allowed participants to answer only the items that concerned them. Thus, questions about behaviors with children, pets, or yard maintenance were not answered by all participants.

**Table 1:** List of Lyme disease prevention behaviors

1 Look into ways to prevent Lyme disease.	
2 Look into potential consequences of Lyme disease for physical or mental health.	
3 Wear pants and long-sleeved sweaters when practicing outdoor activities.	
4 Wear closed shoes when practicing outdoor activities.	
5 Tuck the bottom of sweater or shirt into pants when practicing outdoor activities.	
6 Tuck the bottom of pants into socks or boots when practicing outdoor activities.	
7 Use bug repellent when outdoors.	
8 Walk on cleared paths and trails, avoiding tall grass during outdoor activities.	
9 Wear light-colored clothing to make it easier to check for ticks during outdoor activities.	
10 Examine body for ticks and remove them immediately after being outdoors.	
11 Examine children for ticks and remove them immediately after being outdoors.	
12 Make children take a bath or shower to examine them for ticks and remove them immediately after being outdoors.	
13 Examine clothes and items to avoid bringing ticks into home after being outdoors.	
14 Put clothes in the dryer for six minutes to eliminate ticks that may be there after being outdoors.	
15 Regularly mow lawn or have it mown.	
16 Have a fence around property to prevent deer from coming in the yard.	
17 Increase frequency of lawn maintenance (pick dead leaves, weeds, branches, or twigs).	
18 Have a path or layer of wood chips or mulch to separate the patio, garden, or other installations from the trees or tall grass.	
19 Examine pets for ticks when they come in from outdoors.	

Items 3 through 14 and item 19 were coded according to a five-point scale with options: “Always,” “often,” “occasionally,” “rarely,” and “never.” Those answers were then dichotomized with “Always” and “Often” being considered to be LDPB. Items 1, 2, 16, and 18 were dichotomous and used a yes or no format. Item 15 used a four-point scale with options: “Yes, more than once a week,” “Yes, once a week or less,” “no,” and “I don’t have a lawn.” Item 15 was dichotomized as well, with the first two options being considered preventive. The “I don’t have a lawn” option was considered a missing value. Item 17 was coded with a five-point scale with options: “More than once a week,” “once a week,” “once or twice a month,” “less than once a month,” and “never.” Item 17 was dichotomized, and the first two options (i.e. “always” and “often”) were considered to be preventive. Responses to the items were summed to yield a measure of preventive behaviors (Cronbach’s α = 0.710).

#### Theory of planned behavior constructs

##### Attitude toward LDPB

Responses to the following question were used as reflective indicators of attitude toward the implementation of LDPB: adopting behaviors that will protect me against Lyme disease in the next year will be […]. Participants rated the item on a four-point scale ranging from “very useful” (4) to “very useless” (1).

##### Perceived social norms

Responses to the question were used to measure participants’ perception of social norms related to the implementation of LDPB: if I adopt behaviors to protect myself against tick bites and therefore Lyme disease in the next year, people who are important to me will support my choice. Participants rated the item on a four-point scale ranging from “strongly agree” (4) to “strongly disagree” (1).

##### Perceived behavioral control

Responses to the two questions (correlation: r = 0.398) were used to assess participants’ perceived control over LDPB: (a) it will be easy to protect myself against Lyme disease in the next year, (b) adopting behaviors to protect myself against Lyme disease in the next year will be […]. Participants rated each item on a four-point scale ranging, respectively, from “strongly agree” (4) to “strongly disagree” (1) and from “very easy” (4) to “very difficult” (1). Responses to the items were summed to obtain a measure of moral norms.

##### Intention to adopt LDPB

Intention to adopt LDPB was assessed by these two questions: (a) I intend to adopt behaviors to protect myself against tick bites and Lyme disease in the next year, (b) I have made up my mind to adopt behaviors to protect myself against Lyme disease in the next year. Participants rated each item on a four-point scale ranging from “strongly agree” (4) to “strongly disagree” (1). Responses to the items were summed to yield a measure of preventive behaviors (r = 0.691).

#### Perceived severity and risk

Two variables were added as background factors to the theory of planned behavior: one measured perceived severity of the consequences for health in the event of contracting Lyme disease, and the other measured the perceived risk of contracting Lyme disease in the next year.

##### Perceived severity

Perceived severity of Lyme disease was assessed by one question: “If you were to contract Lyme disease, would you say that the consequences for your health would be very serious?” Participants rated it on a four-point scale ranging from “Yes, absolutely” (4) to “No, not at all” (1).

##### *Perceived* risk

One question was used to measure participants’ perceived risk of contracting Lyme disease: “In your opinion, what is the risk of you contracting Lyme disease in the next year?” Participants rated it on a six-point scale ranging from “Very high” (6) to “Nil” (1).

### Statistical analysis

Before performing any statistical analysis, we reweighted the data so that age, gender, number of people living in the household, education level, income, and proportions of people living in each administrative regions would be the same in the sample and the target population [50]. We also conducted a single imputation to handle the missing data for education level, age, household size, household structure, and income with predictive mean matching [51]. We then conducted a series of analyses to construct and evaluate the validity of the Lyme disease prevention index (Objective 1).

First, an item analysis was performed to analyze the relevance of the items and verify the reliability of the index using item response theory (IRT) and more particularly Samejima’s graded response model [52]. The objective of this item analysis was to assess the ability of the items to differentiate between individuals who adopt Lyme disease prevention behaviors and those who do not, according to a psychometric parameter called item discriminant power. This psychometric parameter could be conceived as a description of the association between each item and the measured construct, Lyme disease prevention behavior in the current study.

The item analysis aimed to determine which items to retain in the final index. Baker [53] proposes the following classification to evaluate the discrimination power of an item: (a) very poor: 0.34 or less; (b) poor: 0.35–0.64; (c) moderate: 0.65–1.34; (d) good: 1.35–1.69; and (e) very good: 1.70 or higher.

Second, we conducted a confirmatory factor analysis (CFA) for the retained items to assess the unidimensionality of the prevention index. We tested a model with a single construct representing Lyme disease prevention measured by the retained behaviors. We used various fit indices to assess the fit of our data with this model. The comparative fit index (CFI) and the Tucker-Lewis index (TLI) indicate a good model fit when they exceed 0.90 or 0.95 and the root mean square error of approximation (RMSEA) indicates a good fit when its value is below 0.08 or 0.06 [54, 55].

Third, to further ascertain the nomological validity of the index (a form of validity that pertains to the testing of proposed relationships among constructs in a model), we tested a model including the Lyme disease index as a dependent variable and the TPB constructs and a measure of perceived severity of Lyme disease and of the perceived risk of contracting Lyme disease in the next year, as determinants of the index. Testing the relationships of the index with constructs of a recognized psychosocial theory can further confirm that the index measures Lyme disease prevention behaviors (LDPB). This also helps to identify the factors that determine the adoption of LDPB and the ones that need reinforcement to support adoption of LDPB. Moreover, this model was tested by structural equation modelling (SEM) using Mplus 8 [56].

Model fit is evaluated using CFI, TLI and RMSEA with the same criterion values as for the confirmatory analysis [54, 55]. For CFI and TLI, acceptable model fit is indicated by values greater than or equal to 0.90 and less than 0.95, and excellent model fit is indicated by values greater than or equal to 0.95. For RMSEA, values between 0.05 and 0.08 indicate adequate model fit, whereas values less than or equal to 0.05 indicate excellent model fit [54, 55].

In some cases, perceived behavioral control also has a moderating effect on the effect of intention on behavior [57]. This interaction between latent variables is also tested once the final model without moderation is obtained and show a good fit. Model fit statistics like CFI, TLI, and RMSEA are not available when models with latent variable interaction are being tested with Mplus with function [58].

## Results

Because of a high rate of missing data, items 11, 12, and 19 were not retained for the following analyses. Missing data rates were 94.03, 94.03, and 65.70%, respectively. These high rates were due to the filter questions retaining only participants with children under 5 years old for items 11 and 12 and only those with pets for item 19. Consequently, the psychometric analyses were performed on the retained 16 behavioral items.

### Descriptive analyses

Means and standard deviations for each variable measured are presented in Table 2. Participants reported adopting a relatively low number of LDPB (M = 4.19 on a maximum score of 10), but a high intention to adopt these behaviors (M = 6.50 on a maximum score of 8), a favorable attitude toward LDPB (M = 3.25 on a maximum score of 4), a high perception of social pressure to perform the behaviors (M = 3.45 on a maximum score of 4), and a strong perception of control over the LDPB (5.59 on a maximum score of 8). The results also showed that people perceived a moderate risk of contracting Lyme disease (M = 3.08 on a maximum score of 6) and a strong risk that Lyme disease would have a negative impact on their health (M = 3.52 on a maximum score of 4). Consistent with many studies [59], the results in Table 2 revealed that the TPB variables were significantly correlated with intentions and the associated behaviors (LDPB in the present study). The results also indicated that the variables (perceived severity and risk) of the health belief model were significantly correlated with intentions and LDPB (see Table 2).

**Table 2:** Descriptive analyses and correlation matrix of TPB variables

Variables	# of Items	*Mean*	SD	1	2	3	4	5	6	7
1 Adaptation index	10	4.19/10	1.88	–						
2 Intention	2	6.50/8	1.32	.389**	–					
3 Attitude	1	3.25/4	0.98	.067**	.272**	–				
4 Social norms	1	3.45/4	0.64	.193**	.548**	.179**	–			
5 Perceived behavioral control	2	5.59/8	1.30	.314**	.234**	.031	.190**	–		
6 Perceived risk	1	3.08/6	1.11	.328**	.261**	.016	.124**	−.105**	–	
7 Perceived severity	1	3.52/4	0.62	.135**	.303**	.083**	.129**	.019	.183**	–

*p < .05 **p < .01

### Item analysis

Excel add-in EIRT [60] provided the results for the item analysis. These results revealed that 14 of the 16 items composing the LDPD scale have acceptable discrimination power (i.e. they can help to differentiate individuals who adapt well to avoid Lyme disease from those who do not adapt as well). “Have a fence around property to prevent deer from coming in the yard” (item 16) and “Have a path or layer of wood chips or mulch to separate the patio, garden, or other installations from the trees or tall grass” (item 18) were removed because of poor correlations with the rest of the items (*ρ* = 0.170 and *ρ* = 0.180, respectively) as well as poor discrimination indices (discrimination power < .34; see the second column in Table 3). It is more likely that respondents adopted those behaviors not to prevent Lyme disease but for landscaping purposes.

**Table 3:** Discrimination power for each preventive behavior

Adaptive behaviors	Discrimination power	99% CI	Mean
1. Look into ways to prevent Lyme disease	1.125	[0.960–1.289]	0.473
2. Look into potential consequences of Lyme disease for physical or mental health	1.177	[1.009–1.345]	0.476
3. Wear pants and long-sleeved sweaters when practicing outdoor activities	1.342	[1.154–1.530]	0.382
4. Wear closed shoes when practicing outdoor activities	1.037	[0.877–1.198]	0.694
5. Tuck the bottom of sweater or shirt into pants when practicing outdoor activities	1.109	[0.921–1.297]	0.239
6. Tuck the bottom of pants into socks or boots when practicing outdoor activities	1.887	[1.564–2.211]	0.106
7. Use bug repellent when outdoors	1.221	[1.034–1.408]	0.299
8. Walk on cleared paths and trails, avoiding tall grass during outdoor activities	0.858	[0.704–1.012]	0.774
9. Wear light-colored clothing to make it easier to check for ticks during outdoor activities	1.147	[0.956–1.338]	0.248
10. Examine body for ticks and remove them immediately after being outdoors	2.567	[2.212–2.922]	0.201
13. Examine clothes and items to avoid bringing ticks into home after being outdoors	2.800	[2.372–3.228]	0.136
14. Put clothes in the dryer for six minutes to eliminate ticks that may be there after being outdoors	1.749	[1.342–2.155]	0.043
15. Regularly mow lawn or have it mown	0.483	[0.356–0.610]	0.802
16. Have a fence around property to prevent deer from coming in the yard	0.221	[0.094–0.347]	0.497
17. Increase frequency of lawn maintenance (pick dead leaves,weeds, branches, or twigs)	0.482	[0.344–0.619]	0.453
18. Have a path or layer of wood chips or mulch to separate the patio, garden, or other installations from the trees or tall grass	0.321	[0.177–0.464]	0.343

The results in Table 3 also showed that the most adopted behavior was “Regularly mow lawn or have it mown” (80.2%) and that the least adopted behavior was “Put clothes in the dryer for six minutes to eliminate ticks that may be there after being outdoors” (4.3%).

### Confirmatory factor analysis

**Fig. 2:**
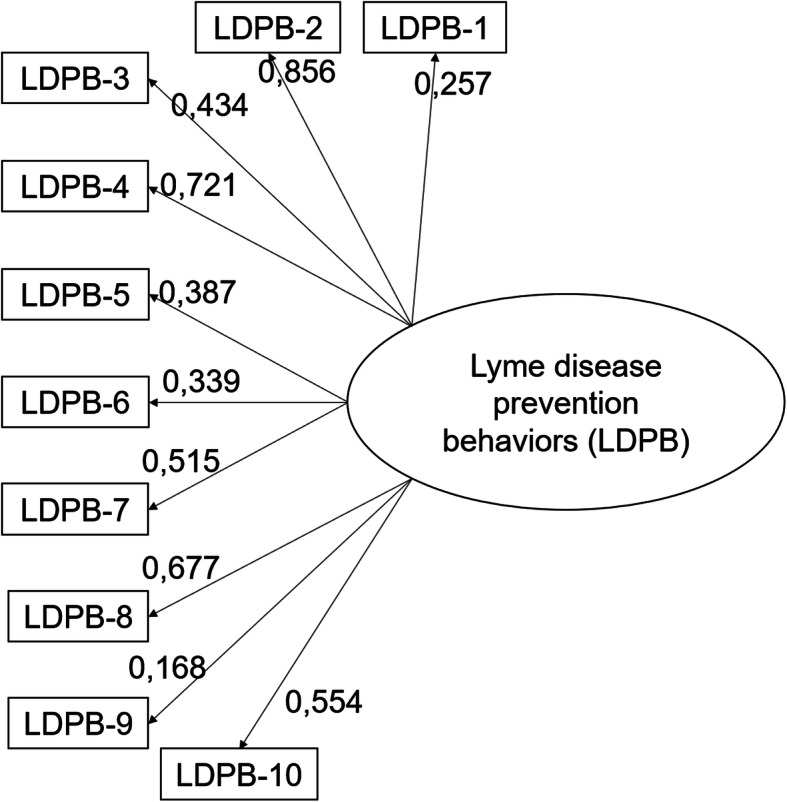
Final model for the Lyme disease prevention index tested by confirmatory analysis. LDPB-1: Look into ways to prevent Lyme disease; LDPB-2: Wear clothes that cover more skin; LDPB-3: Tuck clothes when practicing outdoor activities; LDPB-4: Use bug repellent when outdoor; LDPB-5: Walk on cleared paths and trails, avoiding tall grass during outdoor activities; LDPB-6: Wear light-coloured clothing to make it easier to check for ticks during outdoor activities; LDPB-7: Examine body for ticks and remove them immediately after being outdoor; LDPB-8: Examine clothes and items to avoid bringing ticks into home after being outdoor; LDPB-9: Put clothes in the dryer for 6 minutes to eliminate ticks that may be there after being outdoor; LDPB-10: Increase frequency of lawn maintenance including mowing

The results of the CFA with all 14 remaining items showed a poor fit (CFI = 0.759 TLI = 0.716 and RMSEA = 0.063; χ^2^(77) = 675.698, *p* < 0.0001). Mplus provided modification parameter estimates and indices to identify problematic indicators. Some pairs of items had strong correlations and were thus combined into a single construct. “Look into ways to prevent Lyme disease” and “Look into potential consequences of Lyme disease for physical or mental health” were combined into “Look for information about Lyme disease,” which was set to “yes” if respondents did at least one of the items. “Wear pants and long-sleeved sweaters when practicing outdoor activities” and “Wear closed shoes when practicing outdoor activities” were combined into “Wear clothes that cover more skin,” and the behavior was considered adopted if respondents did at least one of the two former behaviors. “Tuck the bottom of sweater or shirt into pants when practicing outdoor activities” and “Tuck the bottom of pants into socks or boots when practicing outdoor activities” were combined into “Tuck clothes when practicing outdoor activities,” which was also considered adopted if respondents did at least one of the behaviors. “Regularly mow lawn or have it mown” and “Increase frequency of lawn maintenance” were also combined into “Increase frequency of lawn maintenance including mowing,” which was considered adopted if at least one of the two behaviors were adopted. With those changes, the model fit indices indicated an adequate fit (CFI = 0.941, TLI = 0.924, and RMSEA = 0.029). Thus, the final model was comprised of 10 behaviors (a combination of behaviors 1 and 2; 3 and 4; 5 and 6, 7, 8, 9, 10, 13, 14; and 15 and 17). Figure 2 presents this final prevention index.

### Theory of planned behavior

**Fig. 3:**
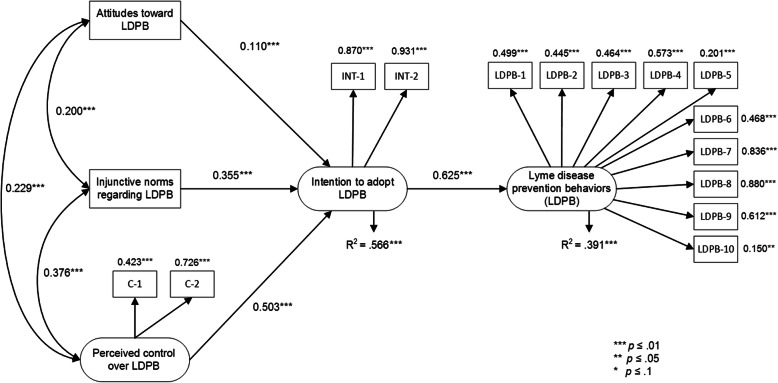
Final TPB model without moderating effect of perceived control on Lyme disease prevention behavior index

The tested TPB model without the moderating effect of perceived control on the behavior index (see Fig. 3) accounted for 56.6% of the intention to adopt LDPB and 39.1% of the variance of the preventive behaviors. Attitudes, social norms, and perceived control all proved to be significantly associated with behavioral intention (γ = 0.110, γ = 0.355, and γ = 0.503, respectively, and all with *p* < 0.001). The association between intention and behavior was significant (γ = 0.625 with *p* < 0.001). The fit of the model was good (CFI = 0.97; TLI = 0.964; RMSEA = 0.028; χ^2^(99) = 253.482, *p* < 0.0001).

In a second analysis, the perceived risk of contracting Lyme disease and the perceived impact of this disease (severity) on health were introduced simultaneously into the standard TPB as background factors. The fit of the model to the data was poorer than that of the standard model, but nonetheless acceptable (CFI = .936; TLI = .921; RMSEA = .038; χ^2^(125) = 469.044, *p* < 0.0001). This model, to which we added two external variables, accounted for 59.4% (+ 2.8%) of the variance of people’s behavioral intention to adopt LDPB and 38.5% (− 0.6%) of the variance of the preventive behaviors. The effect of perceived risk was significant on the perceived social norms (γ = 0.202; *p* < 0.001) and perceived behavioral control (γ = − 0.134; *p* < 0.001). Severity was significant on attitude (γ = 0.134; *p* < 0.001), perceived social norms (γ = 0.239; *p* < 0.001), and perceived behavioral control (γ = 0.116; *p* = 0.006).

**Fig. 4:**
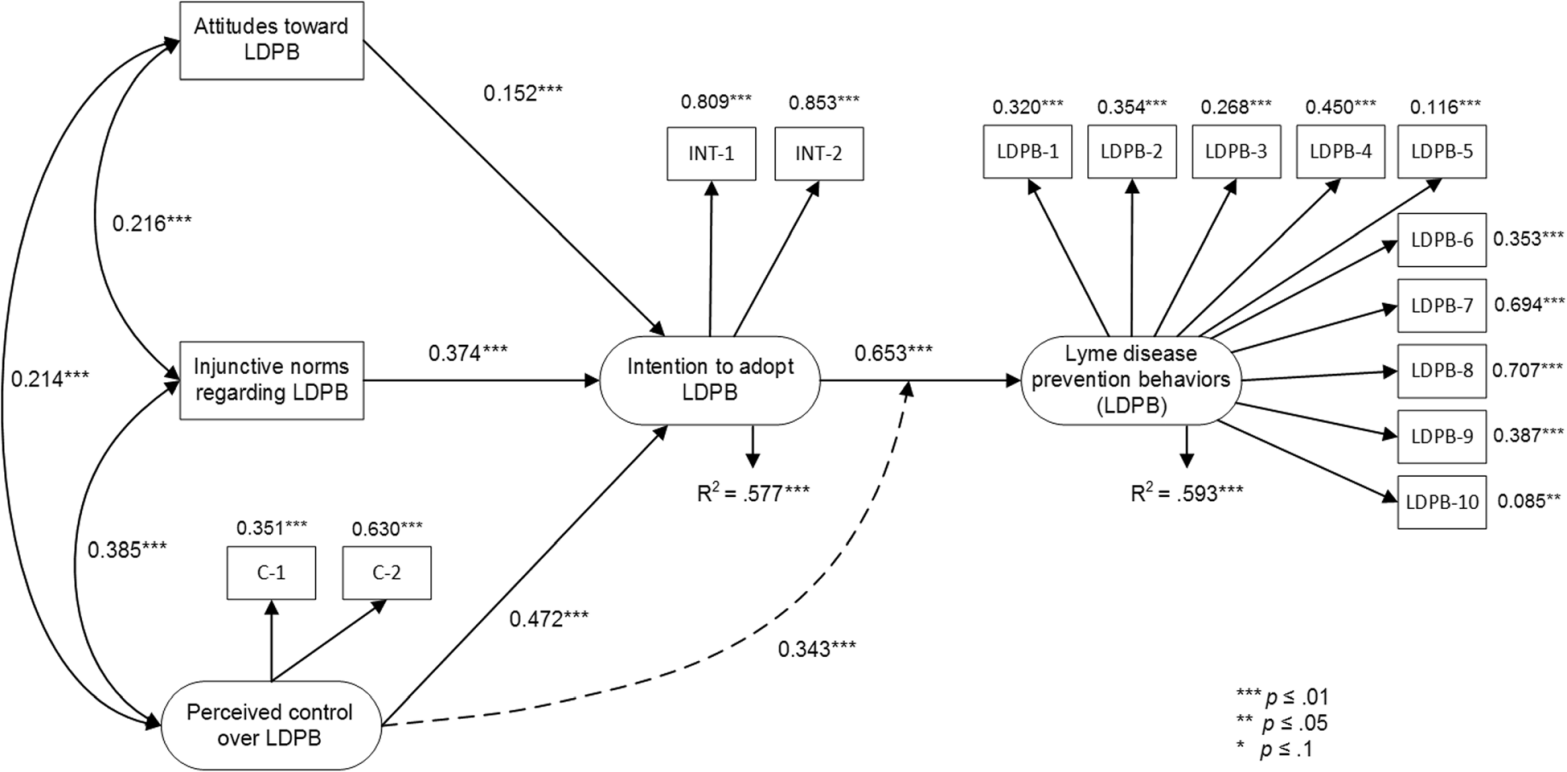
Final TPB model with moderating effect of perceived control on Lyme disease prevention behavior index

In a third analysis, we tested the standard TPB model with the moderating effect of perceived control on the relationship between intention and behavior. We chose that model because it showed better fit indices than the extended model in which we had added two predictive variables (perceived risk and perceived severity). The results revealed that it (see Fig. 4) accounted for 57.7% of the intention to adopt LDPB and 59.3% of the variance of the behaviors. Again, attitude, social norms, and perceived control all proved to be significantly associated with behavioral intention (γ = 0.152, γ = 0.374 and γ = 0.472, respectively, and all with *p* < 0.001). The association of intention with behavior was significant (γ = 0.653 with *p* < 0.001), as was the moderating effect of perceived control on the association of intention with behavior (γ = 0.343 with *p* = 0.01). Model fit indices like CFI, TLI, and RMSEA are not available for testing a model with a moderating effect [58]. Thus, we used the proportion of variance explained in the intention to perform LDPB and the actual adoption of LDPB to compare the TPB model without interaction with the TPB model with interaction. We kept the model with the moderating effect of perceived control on the association between intention and LDPB. We did this because of the greater proportion of variance explained, in line with the TPB, which assesses the presence of this moderating effect [57].

## Discussion

The main goal of this study was to produce and validate an index of adaptation to Lyme disease that could be used to monitor the evolution of the Quebec population’s adoption of preventive behaviors. Concerning the population’s adaptation to Lyme disease, the data from this study show that Quebeckers are eager to learn more about the disease and how to better prepare against it, with 56.90% of respondents seeking information in this regard. Overall, the data from the final index, composed of 10 behaviors, shows that Quebeckers are still at the first stage of adopting preventive behaviors: the rate of adoption of each behavior varies widely, with the least common being “Put clothes in the dryer for six minutes to eliminate ticks that may be there after being outdoors” at only 4.30%, and the most commonly practiced being “lawn maintenance including mowing” at 83.80%. It seems that the more specific LDPB are the least commonly practiced. Indeed, drying the clothes after being outdoors or examining the clothes or the skin for ticks are both adopted by 20% or less of the respondents. Of note, all of the measured behaviors that are comparable to similar behaviors examined by Aenishaenslin et al. [27] showed an increased rate of adoption in our study. As an example, in their 2015 study, where they examined the adoption of LDPB in Québec’s Montérégie region, performing tick checks after outdoor activities was reported by only 7.00% of the participants. Such a behavior could be compared to examining the clothes or the skin in our study, in which the reported adoption rates were 13.60 and 20.01%, respectively. While the specific questions and behaviors used in their study were different from ours, this highlights the importance of creating an index that can be reused throughout the years to monitor the evolution of Quebeckers’ adaptation to Lyme disease, as the infected ticks become more widespread across the province.

On another note, the final index combined some of the behaviors originally included in the study with others that were similar, but also excluded some that had poor fit discrimination indices. For example, we found that most behaviors related to lawn care had poor correlations with the rest of the items. This could mean that respondents adopted those behaviors for general landscaping reasons, not as preventive measures specifically designed to protect themselves from Lyme disease. The final result was an index of adaptation to Lyme disease composed mainly of behaviors closely related to individuals and their own bodies and clothes, instead of more structural behaviors related to their households and lands. These individual behaviors are all easy to adopt, consisting of simple actions like checking for ticks on your clothes or skin after coming back from a hike in the woods, and are commonly suggested by health authorities and even some local municipalities. They are also, and most importantly, good indicators of a population’s rate of adaptation because, unlike some of the behaviors removed from the index because of poor fit, they seem to be more often undertaken specifically to prevent Lyme disease.

Our results also showed that risk perception was positively correlated with the adoption of LDPB. Furthermore, they showed that perceived risk had a significant influence on perceived control and perceived social norms. Perceived risk seemed to have an influence on the adoption of LDPB and needs to be further investigated. Measuring perceived risk as a latent construct with multiple indicators could be a solution to help better estimate its influence in the TPB model of LDPB adoption.

Even though the TPB model including perceived risk and perceived severity as background factors was not the tested model with the highest data fit, the significant relationship observed between risk perception and perceived severity with the adoption of LDPB cannot be dismissed. This finding is also in line with other studies on perceived risk and preventive health behaviors, which have demonstrated that risk perception was associated with the adoption of preventive behaviors [27, 47, 61–63]. As such, while this finding is unsurprising, it provides important information that could be used by health authorities and local governments, who would do well to include information on the risks and consequences of Lyme disease when creating information booklets or messages intended for the population. However, it should be noted that, overall, Lyme disease is already perceived by most of our respondents (94.3%) as having a severe impact on health, and that despite the currently low rate of confirmed cases in Quebec, 34.6% of them perceived a risk of being infected with the disease in the following year.

In this same line of inquiry, we conducted some basic analyses of the decision-making process that could explain why people would choose to adopt LDPB. The TPB model had excellent results, showing that all variables of the model (i.e. attitude, social norms, and perceived control) were significantly associated with intention to adopt preventive behaviors, and that intention itself was significantly associated with adoption of LDPB. While the use of the TPB model in this study was limited to a small subset of questions, further research on the specific variables making up the attitudes, social norms, and perceived control over LDPB could help provide tailored messaging specific to targeted demographics. Such targeted messaging, using the data obtained from the model, has been proven to be more effective than more generic health messages in other cases involving health behaviors [64, 65].

This study has some limitations. First, though it is comparable to most other studies on pro-environmental behavior [30, 66, 67], ours was reliant on self-reported of adoption of preventive behaviors, which could lead some participants to overestimate the extent to which they adopted these behaviors. Consequently, to reduce participants’ socially desirable response bias, we indicated to the participants that their responses were anonymous. Second, the study’s low response rate (24.5%) suggests that the sample may be biased, as the participants were likely to be more interested in adopting Lyme disease prevention behaviors than was the general population. Third, the TPB’s belief variables could not be measured due to concerns about the length of the questionnaire. We preferred to limit the length of the questionnaire to favor a better response rate. Measuring participants’ behavioral, normative and control beliefs could have provided more detailed information regarding the dominant beliefs that underlie people’s decisions to adopt or not Lyme disease prevention behaviors. As previously mentioned, such a study could also help health and government officials to develop more tailored messages in the Province of Quebec.

## Conclusions

Our findings led to the creation and validation of a Lyme disease prevention index made from a specific set of behaviors easily adoptable by people living in areas at risk of Lyme disease. As ticks progress further north, year after year, the widespread adoption of these measures would help protect the population from this disease. Our index can be used by public health agencies, researchers, and professionals to monitor the evolution over time of individuals’ rates of LDPB. Finally, the results of this study also showed the usefulness of the TPB as a framework for understanding the adoption of LDPB and how intention to adopt such behaviors is formed. It explained 57.7% of the variance of intention, along with a high percentage of the effect of intention on behavior (65%), making it a good questionnaire for further studies in this field.

## Supplementary information

**Additional file 1.** Questionnaire.pdf. Questionnaire on Individual Adaptation to Lyme Disease. Description of data: English language version of the questionnaire developed and used for this study.

## Data Availability

The datasets used and/or analyzed during the current study are available from the corresponding author on reasonable request.

## Abbreviations

TPB: Theory of planned behavior
LDPB: Lyme disease prevention behavior
CFA: Confirmatory factor analysis
CFI: Comparative fit index
TLI: Tucker-Lewis index
RMSEA: Root mean square error of approximation
SEM: Structural equation model
