# Supplementary material for: Development and validation of a behavioral index for adaptation to lyme disease
Source: BMC Public Health. 2020 Sep 21;20:1435. doi: 10.1186/s12889-020-09535-2 (PMC7507261; doi:10.1186/s12889-020-09535-2)
Supplement: Supplementary file 1 — Additional file 1. Questionnaire.pdf. Questionnaire on Individual Adaptation to Lyme Disease. Description of data: English language version of the questionnaire developed and used for this study. [file 12889_2020_9535_MOESM1_ESM.pdf]

Questionnaire on Individual Adaptation to Lyme Disease

STREAM 1 - Study 1.5, Measurement Time 1

OQACC

Observatoire québécois
de l'adaptation
aux changements climatiques

Université Laval
Fonds vert
Institut national de santé publique Québec

ENGLISH VERSION

April 30th, 2018

COLLECTION QUESTIONNAIRE FOR STUDY 1.2, TIME 1

Section 1 – Secondary data (to be entered by the interviewer, outside the interview)

LANG

1:
Version of the questionnaire used

French version 01
English version 02

CODE

2:
Six-character postal code

REG

3:
Name of the administrative region:
Estrie 01
Montérégie 02
Mauricie 03
Centre-du-Québec 04
Outaouais 05

VILL

4:
Name of the municipality

SEXE

5:
Respondent's sex
Woman.. 01
Man 02

DATE

6:
Date of the interview
Please write the date in a format that includes the day, the month and the year.

HRE

7:
Duration of the interview
Please write the duration in a format that includes the number of minutes and of seconds.

Section 2 – Recruitment and consent to participate in the study

Reminder - The recruitment criteria are: Respondents must be at least 18 years old, as well as be able to complete an interview and converse in French or in English. It is not necessary to choose a respondent at random (date of next birthday) when more than one person meets the criteria.

(Note to the interviewer: In the following text, do not read the "note to the interviewer" or the italicized text to the respondent.)

Hello! My name is [*interviewer's first name*] and I am with the [*name of the survey firm*] survey firm. I am calling you today as part of a Université Laval study that pertains to Quebeckers' behaviours for adapting to Lyme disease and that is mandated by the Quebec national institute of public health *(*Institut national de santé publique du Québec). Could I please speak to someone living at your address who is 18 years old or over?

(*Note to the interviewer: If the person who answered the phone is the one who continues the interview, go directly to paragraph B. If you speak to a new person, read paragraphs A and B.)*

Paragraph A:

Hello! My name is [*interviewer's first name*] and I am with the [name of the survey firm] survey firm. I am calling you today as part of a Université Laval study that pertains to Quebeckers' behaviours for adapting to Lyme disease and that is mandated by the Quebec national institute of public health *(*Institut national de santé publique du Québec). Before continuing, I would like to make sure that you are indeed 18 years old or over. Is this the case?

(*Note to the interviewer: If so, continue. If not, ask to speak to a person living at that address who is at least 18 years old*).

Paragraph B – Presentation of the study and information on data protection:

We would need your collaboration to answer a questionnaire as part of a study conducted by Université Laval's Québec Observatory of Adaptation to Climate Change. The objective of the study is to document the adoption of various behaviours for preventing Lyme disease. Your answers to this questionnaire are anonymous and will be stored in a secure location. They will be very useful to public health officials in preventing the consequences of Lyme disease for the health of Québec's population.

Verbal statement of consent

Do you agree to participate in this study?

Note to the interviewer:

If the person contacted agrees to participate in the study: Thank the person and continue with the questionnaire.

If the person refuses to participate in the study: Thank the person for his or her time and end the call.

Section 3 – Questionnaire

CM1

1:
Including yourself, how many people live at your address?
Note to the interviewer: Check to be sure that the respondent counted everyone, including those who usually live there but are currently away, like students who come back to live with their parents, spouses working out of town and people temporarily hospitalized.

WRITE THE NUMBER OF PEOPLE: _______

Live alone .... 01 => QUA1
DNK 98
Refusal... 99

CM2

2:
Which of the following types of households best describes your current situation?

You live as a couple, without children .... 01 => QUA1
You live as a couple with one or more children (biological or not)... 02
You live alone with one or more children (biological or not)............................. 03
You live with one or more people who are not related to you (e.g. co-tenant) 04
You live in another type of household 05
DNK 98
Refusal... 99

CM3

3:
Among the children who live at this address, how many are aged…?

Note to the interviewer: *They could be children who do not belong to the respondent, such as grandchildren living with their grandparents or those of a co-tenant.*

READ THE CATEGORIES AND WRITE THE NUMBER OF CHILDREN PER STRATUM

From 0 to 5 years old: ________

From 6 to 12 years old: ________

From 13 to 17 years old: ________

18 years old or over: ________

No children:.. 97
DNK 98
Refusal... 99

QUA1

4:
In all, how many years have you lived in this municipality?

WRITE THE NUMBER OF YEARS: _____ OR “00” IF LESS THAN ONE YEAR
Less than one year 00
DNK 98
REFUSAL 99

CM4

5:
Is it your main residence or a second home?

Main residence 01
Second home.. 02
DNK 98
Refusal.. 99

SUSC01

6:
Do you have access to a yard or an outdoor garden?

Yes.. 01
No 02
DNK 98
Refusal.. 99

LOG1

7:
Is your home less than 150 metres from a location where there are forests, a small wood or tall grass?

NOTE FOR THE INTERVIEWER: SMALL WOOD = A PLACE WHERE THERE IS ONLY SOME TREES (3-4)

Yes.. 01
No 02
DNK 98
Refusal.. 99

The next two questions will deal with outdoor activities commonly practiced between May and October. For your information, by “outdoor activities,” we mean activities practiced in forests, woods, tall grass or on your property.

SUSC02

8:
During your main occupation (work, studies or other), how much time do you spend on average doing outdoor activities?

READ THE CHOICES

Five hours or more per week.. 01
From one to five hours per week 02
Less than one hour per week 03
I do not practice outdoors activities 04
DNK 98
Refusal.. 99

SUSC03

9:
During your recreational activities (camping, outdoor sports, hunting or other), how much time do you spend on average outdoor?

READ THE CHOICES

Five hours or more per week.. 01
From one to five hours per week 02
Less than one hour per week 03
I do not practice recreational activities outdoors 04
DNK 98
Refusal.. 99

I will now ask you a few questions about Lyme disease.

CONN1

10:
Before responding to this survey, had you ever heard of Lyme disease?

Yes.. 01
No 02
DNK 98
Refusal.. 99

CONN2

11:
I am going to read you four descriptions. Please tell me which one you believe best describes Lyme disease.

READ THE CHOICES

It's a disease transmitted through contact with other people 01
It's a disease transmitted through tick bites.. 02
It's a disease transmitted through saliva 03
It's a disease transmitted through a dog bite.. 04
DNK 98
Refusal.. 99

CONN3

12:
Based on your current knowledge, the first symptom of Lyme disease is generally:

READ THE CHOICES

Diarrhea 01
Vomiting... 02
A red plaque on the skin.. 03
Nasal congestion 04
A persistent cough.. 05
DNK 98
Refusal.. 99

CONN4

13:
Have you ever heard of the website eTick.ca ? It is a public platform developed to identify ticks.

LISEZ LES CHOIX

Yes.. 01
No 02
DNK 98
Refusal.. 99

In fact, Lyme disease is transmitted through the bite of a particular species of ticks. Keep this in mind will you answer the following questions.

QUA2

14:
Do you believe that it is possible to contract Lyme disease in your municipality?

Yes.. 01
No 02
DNK .... 98 => RISQ1
Refusal.. 99 => RISQ1

QUA3

15:
Which source(s) did you consult to obtain this information?

READ THE CHOICES

Your municipality 01
Your neighbours 02
Your family and friends.. 03
The media (radio/Web/newspapers/television) 04

Others. Specify 05
DNK 98
Refusal... 99

RISQ1

16:
In your opinion, what is the risk of you contracting Lyme disease in the next year? Would you say that it is:

Very high.. 01
High... 02
Moderate.. 03
Low 04
Very low 05
Nil.. 06
DNK 98
Refusal... 99

RISQ2

17:
Without preventive measures, what is the risk that a resident in your area, child or adult, will contract Lyme disease in the next year?

READ THE CHOICES

Very high.. 01
High... 02
Moderate.. 03
Low 04
Very low 05
Nil.. 06
DNK 98
Refusal... 99

EXP1

18:
Since you've been living at your current address, have you ever received information or a warning about Lyme disease? By "warning", we mean a message sent to you by your municipality, the government, the media or health professionals saying to residents that they are at risk of being in contact with ticks infected with the bacteria.

Yes.. 01 => RISQ3
No .... 02 => SEV1
DNK .... 98 => SEV1
Refusal... 99 => SEV1

RISQ3

19:
Do you agree that, generally, you take this information or these warnings related to Lyme disease seriously?

Strongly agree 01
Somewhat agree.. 02
Somewhat disagree 03
Strongly disagree... 04
DNK 98
Refusal... 99

SEV1

20:
If you were to contract Lyme disease, would you say that the consequences for your health would be very serious?

READ THE CHOICES

Yes, absolutely 01
Yes, mostly 02
No, not really... 03
No, not at all.. 04
DNK 98
Refusal... 99

INQ1

21:

Generally, how concerned are you about contracting Lyme disease in the next year?

READ THE CHOICES

Very concerned.. 01
Rather concerned 02
Not very concerned.. 03
Not at all concerned 04
DNK 98
Refusal... 99

SEFF1

22:

Do you agree that it will be easy to protect yourself against Lyme disease in the next year?

READ THE CHOICES

Strongly agree 01
Somewhat agree.. 02
Somewhat disagree 03
Strongly disagree... 04
DNK 98
Refusal... 99

EXP2

23:

Have you ever found a tick on yourself?

Yes... 01
No 02
Uncertain (I think so / don't think so, but I'm not sure) 03
DNK 98
Refusal... 99

EXP3

24:

Have you ever been bitten by a tick?

Yes... 01
No 02
Uncertain (I think so / don't think so, but I'm not sure) 03
DNK 98
Refusal... 99

EXP4

25:

Have any people in your circle ever found a tick on themselves?

Yes... 01
No 02
Uncertain (I think so / don't think so, but I'm not sure) 03
DNK 98

Refusal.. 99

EXP5

26:

Has anyone in your circle ever been bitten by a tick?

Yes... 01
No 02
Uncertain (I think so / don't think so, but I'm not sure) 03
DNK 98
Refusal.. 99

EXP6

27:
Has a doctor ever diagnosed you with Lyme disease?

Yes... 01 => EXP7
No .... 02 => EXP10
Uncertain (I think so / don't think so, but I'm not sure) .... 03 => EXP10
DNK .... 98 => EXP10
Refusal.. 99 => EXP10

EXP7

28:
If EXP6 = 01: When were you diagnosed with Lyme disease?

2018 01
2017 02
2016 03
2015 04
Before 2015 05
DNK 98
Refusal.. 99

EXP8

29:
If EXP6 = 01: Where did you get diagnosed with Lyme disease?

READ THE CHOICES

In Québec 01
In another Canadian province 02
In the United States 03
In another country (specify) 04
DNK 98
Refusal.. 99

EXP9

30:
If EXP6 = 01: Where did you come in contact with Lyme disease?

READ THE CHOICES

In Québec 01
In another Canadian province 02
In the United States 03
In another country (specify) 04
DNK 98
Refusal.. 99

EXP10

31:
Has a health professional diagnosed anyone in your circle with Lyme disease?

Yes 01
No 02
Uncertain (I think so / don't think so, but I'm not sure) 03
DNK 98
Refusal 99

The next questions pertain to the various measures that you can take to protect yourself against Lyme disease, as well as to your perception of their effectiveness.

ADAP1

32:
Have you ever looked into ways to prevent Lyme disease?

Yes 01
No 02
DNK 98
Refusal 99

ADAP2

33:
Have you ever looked into the potential consequences of Lyme disease for your physical or mental health?

Yes 01
No 02
DNK 98
Refusal 99

FILTREA00

The next questions will deal with outdoor activities practiced between May and October. For your information, we are talking here about activities practiced in forests, woods, tall grass or on your property.

If SUSCO02 or SUSCO03 = {04, 98, 99} => FILTREA01

ADAP3

34:
When practicing outdoor activities, do you wear long pants and a long-sleeved sweater?

READ THE CHOICES

Always 01
Often 02
Occasionally 03
Rarely 04
Never 05
DNK 98
Refusal 99

EFF1

35:
In your opinion, how effective is this behaviour for protecting yourself against tick bites?

READ THE CHOICES

Very effective 01
Rather effective 02
Not very effective 03
Not effective 04
DNK 98
Refusal 99

ADAP4

36:

When practicing outdoor activities, do you wear closed shoes?

READ THE CHOICES

Always.......... 01
Often 02
Occasionally 03
Rarely.......... 04
Never 05
DNK 98
Refusal.......... 99

EFF2

37:
In your opinion, how effective is this behaviour for protecting yourself against tick bites?

READ THE CHOICES

Very effective 01
Rather effective.......... 02
Not very effective 03
Not effective.......... 04
DNK 98
Refusal.......... 99

ADAP5

38:
When practicing outdoor activities, do you tuck the bottom of your sweater or of your shirt into your pants?

READ THE CHOICES

Always.......... 01
Often 02
Occasionally 03
Rarely.......... 04
Never 05
DNK 98
Refusal.......... 99

EFF3

39:
In your opinion, how effective is this behaviour for protecting yourself against tick bites?

READ THE CHOICES

Very effective 01
Rather effective.......... 02
Not very effective 03
Not effective.......... 04
DNK 98
Refusal.......... 99

ADAP6

40:
When practicing outdoor activities, do you tuck the bottom of your pants into your socks or boots?

READ THE CHOICES

Always.......... 01
Often 02
Occasionally 03
Rarely.......... 04
Never 05
DNK 98

Refusal.. 99

EFF4

41:
In your opinion, how effective is this behaviour for protecting yourself against tick bites?

READ THE CHOICES

Very effective 01
Rather effective... 02
Not very effective 03
Not effective.. 04
DNK 98
Refusal... 99

ADAP7

42:
When outdoors, do you use a bug repellent (containing DEET, icaridin or picaridin) on your clothes or the exposed parts of your body?

READ THE CHOICES

Always.. 01
Often 02
Occasionally 03
Rarely.. 04
Never 05
DNK 98
Refusal... 99

EFF5

43:
In your opinion, how effective is this behaviour for protecting yourself against tick bites?

READ THE CHOICES

Very effective 01
Rather effective... 02
Not very effective 03
Not effective.. 04
DNK 98
Refusal... 99

ADAP8

44:
When practicing outdoor activities, do you walk on cleared paths and trails, avoiding tall grass?

READ THE CHOICES

Always.. 01
Often 02
Occasionally 03
Rarely.. 04
Never 05
DNK 98
Refusal... 99

EFF6

45:
In your opinion, how effective is this behaviour for protecting yourself against tick bites?

READ THE CHOICES

Very effective 01
Rather effective... 02

Not very effective 03
Not effective 04
DNK 98
Refusal 99

ADAP9

46:
When practicing outdoor activities, do you wear light-coloured clothing to make it easier to check for ticks?

READ THE CHOICES

Always 01
Often 02
Occasionally 03
Rarely 04
Never 05
DNK 98
Refusal 99

EFF7

47:
In your opinion, how effective is this behaviour for protecting yourself against tick bites?

READ THE CHOICES

Very effective 01
Rather effective 02
Not very effective 03
Not effective 04
DNK 98
Refusal 99

ADAP10

48:
After being outdoors, do you examine your body for ticks and remove them immediately?

READ THE CHOICES

Always 01
Often 02
Occasionally 03
Rarely 04
Never 05
DNK 98
Refusal 99

EFF8

49:
In your opinion, how effective is this behaviour for protecting yourself against Lyme disease?

READ THE CHOICES

Very effective 01
Rather effective 02
Not very effective 03
Not effective 04
DNK 98
Refusal 99

IF CM3 [0 à 5 years] ≥ 1 => ADAP11

IF CM3 [0 to 5 years] < 1 => ADAP13

ADAP11

50:
After the children have gone outside, do you examine them for ticks and remove them immediately?

READ THE CHOICES

Always.. 01
Often 02
Occasionally 03
Rarely.. 04
Never 05
DNK 98
Refusal.. 99

EFF9

51:
In your opinion, how effective is this behaviour to protect them against Lyme disease?

READ THE CHOICES

Very effective 01
Rather effective... 02
Not very effective 03
Not effective.. 04
DNK 98
Refusal.. 99

ADAP12

52:
After the children have gone outside, do you make them take a bath or a shower to examine them for ticks and remove them immediately?

READ THE CHOICES

Always.. 01
Often 02
Occasionally 03
Rarely.. 04
Never 05
DNK 98
Refusal.. 99

EFF10

53:
In your opinion, how effective is this behaviour to protect them against Lyme disease?

READ THE CHOICES

Very effective 01
Rather effective... 02
Not very effective 03
Not effective.. 04
DNK 98
Refusal.. 99

ADAP13

54:
After being outdoors, do you examine your clothes and the items that you had with you avoid bringing ticks into your home?

READ THE CHOICES

Always.. 01
Often 02
Occasionally 03

Rarely 04
Never 05
DNK 98
Refusal 99

EFF11

55:
In your opinion, how effective is this behaviour for protecting yourself against tick bites?

READ THE CHOICES

Very effective 01
Rather effective 02
Not very effective 03
Not effective 04
DNK 98
Refusal 99

ADAP14

56:
After being outdoors, do you put your clothes in the dryer for six minutes to eliminate ticks that may be there?

READ THE CHOICES

Always 01
Often 02
Occasionally 03
Rarely 04
Never 05
DNK 98
Refusal 99

EFF12

57:
In your opinion, how effective is this behaviour for protecting yourself against tick bites?

READ THE CHOICES

Very effective 01
Rather effective 02
Not very effective 03
Not effective 04
DNK 98
Refusal 99

The next questions pertain to the various measures concerning your property and your immediate environment that may protect yourself against Lyme disease.

FILTRE01

58:
If SUSC01 = 01: Do you own a yard or an outdoor garden or are you responsible for maintaining one?

IF SUSCO1 ≠ 01 => FILTREA02

READ THE CHOICES

Yes .... 01 => ADAP15
No .... 02 => FILTREA02
DNK .... 98 => FILTREA02
Refusal .... 99 => FILTREA02

ADAP15

59:

Do you regularly mow your lawn or have it mown?

READ THE CHOICES

Yes, more than once a week 01
Yes, once a week or less 02
No 03
I don't have a lawn 04
DNK 98
Refusal 99

EFF13

60:
In your opinion, how effective is this behaviour for protecting yourself against tick bites?

READ THE CHOICES

Very effective 01
Rather effective 02
Not very effective 03
Not effective 04
DNK 98
Refusal 99

ADAP16

61:
Is there a fence around your property to prevent deer from coming into your yard?

Yes 01
No 02
DNK 98
Refusal 99

EFF14

62:
In your opinion, how effective is this behaviour for protecting yourself against tick bites?

READ THE CHOICES

Very effective 01
Rather effective 02
Not very effective 03
Not effective 04
DNK 98
Refusal 99

ADAP17

63:
How often do you maintain your lawn, for example pick up dead leaves, weeds, branches or twigs, or have them picked up? (other than mowing your lawn)

READ THE CHOICES

More than once a week 01
Once a week 02
Once or twice a month 03
Less than once a month 04
Never 05
DNK 98
Refusal 99

EFF15

64:
In your opinion, how effective is this behaviour for protecting yourself against tick bites?

READ THE CHOICES

Very effective 01
Rather effective.. 02
Not very effective 03
Not effective.. 04
DNK 98
Refusal... 99

ADAP18

65:
In your yard, is there a path or a layer of wood chips or mulch to separate your patio, garden or other installation from the trees or tall grass?

READ THE CHOICES

Yes... 01
No 02
DNK 98
Refusal... 99

EFF16

66:
In your opinion, how effective is this behaviour for protecting yourself against tick bites?

READ THE CHOICES

Very effective 01
Rather effective.. 02
Not very effective 03
Not effective.. 04
DNK 98
Refusal... 99

FILTREA02

67:
Do you have any pets that have access to the outdoors?

READ THE CHOICES

Yes... 01 => ADAP19
No .... 02 => INT00
DNK .... 98 => INT00
Refusal... 99 => INT00

ADAP19

68:
When your pets (dog, cat or other) come in from outdoors, do you examine them for ticks?

READ THE CHOICES

Always.. 01
Often 02
Occasionally 03
Rarely... 04
Never 05
DNK 98
Refusal... 99

EFF17

69:
In your opinion, how effective is this behaviour for protecting yourself against tick bites?

READ THE CHOICES

Very effective 01

Rather effective.. 02
Not very effective 03
Not effective.. 04
DNK 98
Refusal.. 99

INT00

The following questions pertain to the behaviours that we just asked you about and that could help prevent tick bites and therefore Lyme disease. We would now like to know your degree of agreement or disagreement with certain statements regarding these behaviours:

INT1

70:
You intend to adopt behaviours to protect yourself against tick bites and Lyme disease in the next year. Do you:

READ THE CHOICES

Strongly agree 01
Somewhat agree.. 02
Somewhat disagree 03
Strongly disagree... 04
DNK 98
Refusal.. 99

NORM1

71:
If you adopt behaviours to protect yourself against tick bites and therefore Lyme disease in the next year, people who are important to you will support your choice. Do you:

READ THE CHOICES

Strongly agree 01
Somewhat agree.. 02
Somewhat disagree 03
Strongly disagree... 04
DNK 98
Refusal.. 99

CONT1

72:
You feel capable of adopting behaviours to protect yourself against Lyme disease in the next year. Do you:

READ THE CHOICES

Strongly agree 01
Somewhat agree.. 02
Somewhat disagree 03
Strongly disagree... 04
DNK 98
Refusal.. 99

INT2

73:
You have made up your mind to adopt behaviours to protect yourself against Lyme disease in the next year. Do you:

READ THE CHOICES

Strongly agree 01
Somewhat agree.. 02
Somewhat disagree 03
Strongly disagree... 04
DNK 98
Refusal.. 99

NORM2

74:
The people who are important to you will adopt behaviours to protect themselves against Lyme disease in the next year. Do you:

READ THE CHOICES

Strongly agree 01
Somewhat agree 02
Somewhat disagree 03
Strongly disagree 04
DNK 98
Refusal 99

We would also like to know if you think that…

ATT1

75:
Adopting behaviours that will protect you against Lyme disease in the next year will be:

READ THE CHOICES

Very useless 01
Slightly useless 02
Slightly useful 03
Very useful 04
DNK 98
Refusal 99

ATT2

76:
Adopting behaviours that will protect you against Lyme disease in the next year will be:

READ THE CHOICES

Very uncomfortable 01
Slightly uncomfortable 02
Slightly comfortable 03
Very comfortable 04
DNK 98
Refusal 99

CONT2

77:
Adopting behaviours to protect yourself against Lyme disease in the next year will be:

READ THE CHOICES

Very easy 01
Slightly easy 02
Slightly difficult 03
Very difficult 04
DNK 98
Refusal 99

VACC00

The following questions are about your degree of agreement or disagreement with certain statements regarding an eventual Lyme disease vaccine.

VACC01

78:
If a vaccine against Lyme disease was available, you would get vaccinated. Do you:

READ THE CHOICES

Strongly agree 01
Somewhat agree.. 02
Somewhat disagree 03
Strongly disagree.. 04
DNK 98
Refusal.. 99

SI CM3 [0 à 5 ans] ≥ 1 => VACC02
SI CM3 [0 à 5 ans] < 1 => VACC03

VACC02

79:
If a vaccine against Lyme disease was available, you would get your child vaccinated. Do you:

READ THE CHOICES

Strongly agree 01
Somewhat agree.. 02
Somewhat disagree 03
Strongly disagree.. 04
DNK 98
Refusal.. 99

VACC03

80:
Vaccines are a danger to your health. Do you:

READ THE CHOICES

Strongly agree 01
Somewhat agree.. 02
Somewhat disagree 03
Strongly disagree.. 04
DNK 98
Refusal.. 99

SOC00

In closing, I will ask you a few questions that will be used only to classify the answers according to each respondent's profile.

AGE1

81:
Could you please tell me how old you are?

WRITE THE AGE HERE: ___________

Note to the interviewer: If the participant refuses, ask if he or she would agree to say in which of the following age ranges he or she falls.

READ THE CATEGORIES

18–24 01
25–29 02
30–34 03
35–39 04
40–44 05
45–49 06
50–54 07
55–59 08
60–64 09

65–69 10
70–74 11
75–79 12
80–84 13
85–89 14
90 and over 15
DNK 98
Refusal 99

NAT1

82:
Were you born…

In Québec .... 01 => SCO1
Elsewhere in Canada .... 02 => SCO1
In a country other than Canada .... 03 => NAT2
DNK 98
Refusal 99

NAT2

83:
In total, how many years have you lived in Québec, since you first arrived?

READ THE CATEGORIES

Less than 1 year 01
From 1 to 4 years 02
From 5 to 9 years 03
10 years or more 04
DNK 98
Refusal 99

SCO1

84:
What is the highest education level you have achieved?

SPONTANEOUS ANSWER. Write down the response
No diploma 01
Elementary school 02
Partial secondary studies (Sec I to IV) 03
High school diploma (Sec V or 12th grade) 04
Partial studies in CEGEP or in a trade or vocational school 05
Diploma or certificate of college, trade or vocational studies 06
Partial university studies 07
University degree 08
Other, specify 09
DNK 98
Refusal 99

ECO1

85:
How would you describe your main occupation? Would you say that you are:
READ THE CHOICES

A full-time worker (salaried or self-employed) 01
A part-time worker (salaried or self-employed) 02
A student 03
Retired or an annuitant 04
On long-term sick leave 05
Receiving employment insurance (including maternity leave) 06
Receiving social assistance (income security) 07
A homemaker 08
Other, specify 09

DNK 98
Refusal... 99

ECO2

86:
At what do you estimate the annual gross income (before deductions) of your household, including all sources of income (pension, salaries, scholarship, etc.)?

READ THE CATEGORIES

Less than $10,000 01
Between $10,000 and $20,000.. 02
Between $20,001 and $30,000.. 03
Between $30,001 and $40,000.. 04
Between $40,001 and $50,000.. 05
Between $50,001 and $60,000.. 06
Between $60,001 and $70,000.. 07
Between $70,001 and $80,000.. 08
Between $80,001 and $90,000.. 09
Between $90,001 and $100,000.. 10
More than $100,000.. 11
DNK 98
Refusal... 99

ECO3

87:
If CM1 ≠ 01: Including yourself, how many people contributed to this annual income in the past year?

WRITE THE NUMBER OF PEOPLE: _________
DNK 98
Refusal... 99

TELWEB1

88:
Do you have access to a landline in your home?

Yes.. 01
No 02

TELWEB2

89:
Do you have Internet access at home?

Yes.. 01
No 02

COOR1

90:
For public health reasons, it is important to monitor the evolution of Quebeckers' adaptation to Lyme disease over time. If there were to be a second phase to this study in about three years, would you accept that we contact you to participate?

Yes.. 01 => COOR2
No .... 02 => End of the interview

COOR2

91:
At what email address or phone number could we contact you? Once again, rest assured that this information will remain strictly confidential and will not be used for any other purpose.

WRITE THE EMAIL ADDRESS: _______________________________

WRITE THE PHONE NUMBER: ______________________

Thank the respondent. End the interview.
